# A new type of congenital hepatic hemangioma - rapid postnatal proliferation followed by regression

**DOI:** 10.3389/fonc.2025.1508461

**Published:** 2025-07-02

**Authors:** Luyao Yang, Weitao Dou, Xin Chen, Jianbo Teng, Xinhong Wei

**Affiliations:** ^1^ Department of Ultrasound, Shandong Provincial Hospital Affiliated to Shandong First Medical University, Jinan, Shandong, China; ^2^ Department of Medical Intervention, Shandong Provincial Hospital, Cheeloo College of Medicine, Shandong University, Jinan, Shandong, China; ^3^ Department of Medical Intervention, Shandong Provincial Hospital Affiliated to Shandong First Medical University, Jinan, Shandong, China; ^4^ Departments of Radiology, Shandong Provincial Hospital Affiliated to Shandong First Medical University, Jinan, Shandong, China

**Keywords:** congenital hepatic hemangiomas, prenatal diagnosis, ultrasonography, magnetic resonance imaging, vascular tumors

## Abstract

**Objective:**

This study aimed to analyze and summarize the prenatal and postnatal imaging findings, as well as the postnatal growth curves, of patients with hepatic hemangiomas (HHs) that were identified *in utero* and continued to proliferate after birth.

**Methods:**

A retrospective study was conducted to collect and analyze data from children with congenital hepatic hemangiomas (CHH) who were diagnosed and followed-up at our hospital between January 1, 2016 and December 30, 2023. These children exhibited rapid postnatal proliferation of lesions, followed by spontaneous regression. The study recorded the patients’ general clinical information, laboratory test results, and pre- and postnatal imaging characteristics of the tumor, as well as changes in tumor volume over time.

**Results:**

Eight patients (four males and four females) were included in this group, with and average gestational age of 37 weeks at the initial onset. The imaging features of this type of hepatic hemangioma are almost indistinguishable from those previously described for CHH. The only difference was that 87.5% of the tumors were located in the left lobe of the liver, and no calcification was observed within the tumors during the prenatal and proliferative stages. The postnatal growth trend of the tumors was very rapid, with complete proliferation occurring within the first six months after birth (median, 66 days) and the peak volume exceeding 1.5 × the initial volume. Of the tumors, 87.5% (7/8) regressed to 80% of the initial volume within one year, and the median time to complete regression was 365 days (range 300-730). None of the patients experienced adverse symptoms or complications during the study period.

**Conclusions:**

This article describes a special type of CHH that can continue to proliferate after birth. However, the tumor spontaneously regresses over time without complications. Therefore, for postnatal CHH growth, regular imaging observation without drug treatment or surgery is recommended, thereby preventing overtreatment while ensuring normal child development.

## 1 Introduction

Congenital hepatic hemangioma (CHH) is the formation of a tumor due to excessive proliferation of vascular endothelial cells, which is currently believed to initiate growth *in utero* and fully develop at birth (1). CHHs are mostly focal lesions, with the majority being asymptomatic, and are typically discovered during routine prenatal examinations (2). The prenatal ultrasound (US) and magnetic resonance imaging (MRI) manifestations of CHH have been summarized in the literature (2–5) and often reveal a focal solid mass with well-defined borders and an uneven low echo, along with multiple vascular echoes and occasional calcification. There was no difference between antenatal and postnatal MR masses, which were often solid and uneven, exhibiting a high signal on T2-weighted (T2-WI) and a low signal on T1-weighted (T1-WI), with flow gaps frequently appearing in high-flow vessels within the lesion.

Based on different clinical regression cycles, they are currently categorized into three types: rapidly involuting congenital hemangioma (RICH), non-involuting congenital hemangioma (NICH), and partially involuting congenital hemangioma (PICH). RICH regress completely within 12–18 months, NICH grow in proportion to body growth, and PICH initially show regression similar to that of RICH but eventually stop regressing (1, 6, 7).

The discovery of a potential type of CHH during follow-up revealed distinct growth cycles and clinical characteristics compared to those of the existing types, including rapid postnatal tumor enlargement (excluding intratumoral bleeding), followed by regression. This paper reviews eight cases of this type of liver congenital hemangioma in children, examining US and MRI manifestations, as well as postnatal growth curves of the tumors, to enhance clinicians’ understanding and facilitate treatment planning.

## 2 Methods

Data of all patients diagnosed with CHH by prenatal imaging at Shandong Provincial Hospital or referred to other hospitals between January 1, 2016 and December 30, 2023 were retrospectively collected. All CHHs were definitively diagnosed using the International Society for the Study of Vascular Anomalie (ISSVA) classification system, employing a multimodal diagnostic approach that integrates clinical manifestations, imaging features, and/or histopathological confirmation, when indicated. The inclusion criteria were as follows: (1) prenatal identification of hepatic mass lesions, (2) definitive CHH diagnosis as per the ISSVA classification guidelines, and (3) postnatal documentation of tumor volume exceeding 1.5 times the initial measurement at disease presentation. The exclusion criteria included cases demonstrating histopathological or clinical discordance with CHH diagnostic standards, no postnatal tumor progression (defined as static or regressive tumor volume dynamics), and patients undergoing postoperative therapeutic interventions or who were lost to follow-up. This study adhered to the principles of the Declaration of Helsinki and was approved by the Ethics Committee of Shandong Provincial Hospital (NO.2019-001). Written informed consent was obtained from all patients and/or their legal guardians (s) prior to registration.

All participants underwent standardized prenatal and postnatal imaging evaluations using US or MRI. Prenatal assessments were conducted using GE Voluson E10 (GE Healthcare, Zipf, Austria) and Samsung W10 (Samsung Healthcare, Gangwon-do, South Korea) ultrasound systems equipped with abdominal transducers (3.5-5.0 MHz). Upon initial diagnosis of a fetal hepatic mass detection, the maternal age and gestational age were documented. Lesion characteristics, including anatomical location, maximum dimensions, margination, and echotexture (homogeneous/heterogeneous), were systematically analyzed using B-mode imaging with a comparative assessment of echogenicity relative to the normal hepatic parenchyma. Specific attention has been directed toward identifying calcifications and venous lakes, which are characterized by irregular vascular dilatations (8). Cardiothoracic ratio quantification was performed, with values exceeding 0.33 considered pathological based on established normative thresholds (<0.33) (9). Color Doppler flow imaging (CDFI) enabled vascular characterization through (2, 10–12) vascular density grading (low: <2 vessels/cm²; moderate: 2–5 vessels/cm²; high: >5 vessels/cm²); feeding vessel identification (portal venous vs. combined portal-hepatic arterial supply); detection of portal-systemic venous shunts (an abnormal connection between a branch of the portal vein and the hepatic vein within a tumor) and vascular dilatation (hepatic veins/arteries); and assessment of peripheral rim vascularity (yes/no). Hemodynamic monitoring included middle cerebral artery peak systolic velocity (MCA-PSV >1.5, multiples of the median [MoM] indicating fetal anemia) and ductus venosus waveform analysis (abnormal A-wave reversal). Postnatal re-evaluations employed identical GE Voluson E10 protocols. All imaging interpretations were performed by sonographers with >10 years of specialized experience, and discordant cases were resolved through a senior consultant.

Fetal and infant MRI examinations were performed using a 1.5 T MAGNETOM Amira (Siemens Shenzhen Magnetic Resonance, Ltd., Shenzhen, China) with a 13-channel body coil in combination with a spine coil. Comprehensive MR characterization was independently conducted by two subspecialty radiologists. Evaluated parameters encompassed: lesion location (left/right lobe distribution); morphological subtype classification (focal/multifocal/diffuse); boundary demarcation clarity; volumetry; signal characteristics on T1WI and T2WI sequences; intralesional hemorrhage/necrosis (binary assessment); peripheral contrast enhancement patterns; hepatic vasculature analysis (normality/dilatation of hepatic arteries, veins, and portal system); presence of portosystemic shunting; and cardiothoracic ratio quantification. Interobserver consensus was achieved through joint reevaluation of discordant cases. The calculation formula of tumor volume refers to the ellipse: a×b×c×0.523 (5).

Demographic documentation included patient gender and serial laboratory evaluations comprising complete blood count (anemia defined as hemoglobin <100 g/L), hepatic function panel, thyroid profile (hypothyroidism criteria: free triiodothyronine <1.78 pg/mL with thyroid-stimulating hormone >7.31 μIU/mL), coagulation studies (fibrinogen <2 g/L indicating coagulopathy), and alpha-fetoprotein (AFP) surveillance (abnormal threshold >25 ng/mL beyond 3 months postnatal) (13). Longitudinal imaging surveillance incorporates detailed lesion characterization and volumetric analysis through serial examinations, with tumor growth kinetics graphically represented using longitudinal growth curves. This multimodal monitoring protocol ensures comprehensive evaluation of lesion progression and systemic complications.

Graphics and calculations in this study were performed with GraphPad Prism software, version 9.0 (GraphPad software, San Diego, CA).

## 3 Result

**Figure 1:**
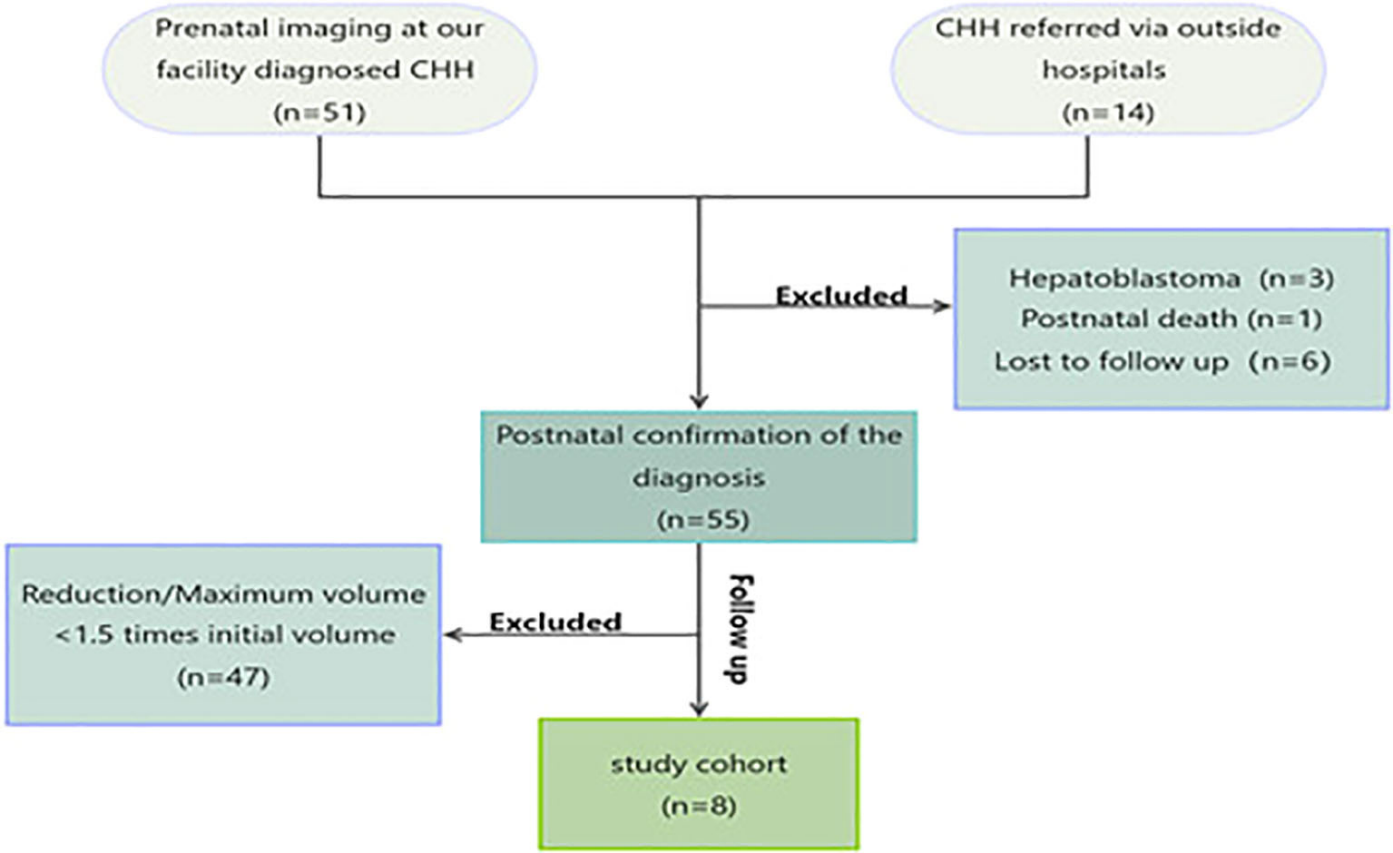
The flowchart.

Finally, eight neonates with CHH who continued to proliferate after birth were enrolled in this study. This process is summarized in **Figure 1**. All the patients underwent prenatal and intrapartum ultrasound surveillance. Prenatal MRI acquisition was completed in four cases (50%). Non-completion causes included voluntary abandonment by pregnant women (3/8, 7.5%) and failed screening due to claustrophobia (1/8, 12.5%).

### 3.1 Prenatal ultrasound features

The average gestational age at the initial occurrence of these 8 cases was 37 weeks + (ranging from 36 to 39 weeks), and all the tumors initially emerged in the late stage of pregnancy; 87.5% (7/8) of the lesions were located in the left lobe of the liver. All lesions were focal and had clear boundaries, with low echogenicity being predominant in 87.5% (7/8) of the lesions, while one lesion was predominantly isoechoic. Honeycomb-like mixed echoes were observed in 75% (6/8) of the lesions, and tubular vascular echoes could be seen within the lesions. No strong calcification-like echoes were observed in any lesions. The median initial volume of the lesions was approximately 33.4 ml (range: 0.3-46.5 ml), with five cases presenting as giant hemangiomas (diameter >4 cm) (14). Additionally, during CDFI observation, 87.5% (7/8) of the lesions exhibited medium-to-high vascularity, with one lesion (No. 4) showing low vascularity; all tracked scans revealed portal vein blood supply to the tumors, and in two cases (No. 5 and No. 7), hepatic artery blood supply near the lesions was also observed. Vascular dilation around the lesions was noted in 37.5% (3/8) of patients. No venous lakes or shunts were observed within the tumors. Peripheral venous flow exhibited a circular or semicircular pattern in 75% (6/8) of patients. All patients had normal cardiopulmonary ratios and venous ductogram blood flow spectra. Detailed data are provided in **Table 1**.

**Table 1:** Ultrasound characteristics of the initial prenatal examination of 8 patients.

Case number	Sex	GA at diagnosis (weeks)	Lesion location	Initial volume (ml)	Echogenicity	Margin	Calcification	Vascularity	Tumor peripheral blood flow	Blood supply vessels	Hepatic vein dilatation	Hepatic artery dilation
1	F	38w+	Left	36.88	Predominantly hypoechoic	well-defined	No	marked	Yes	portal vein	No	No
2	F	37w+	Left	32.42	Predominantly hypoechoic	well-defined	No	marked	Yes	portal vein	No	No
3	M	39w+	Left	43.23	Predominantly hypoechoic	well-defined	No	marked	Yes	portal vein	Yes	No
4	F	39W+	Right	0.30	Uniformly hypoechoic	well-defined	No	mild	Yes	portal vein	No	No
5	F	36W+	Left	10.95	Predominantly hypoechoic	well-defined	No	moderate	Yes	Portal vein + hepatic artery	No	Yes
6	M	39w+	Left	46.48	Predominantly hypoechoic	well-defined	No	marked	Yes	portal vein	Yes	No
7	M	36w+	Left	34.35	Predominantly isoechoic	well-define	No	moderate	Yes	Portal vein + hepatic artery	Yes	Yes
8	M	35w+	left	2.67	Uniformly hypoechoic	well-defined	No	moderate	Yes	Portal vein	No	No

### 3.2 Prenatal MR features

**Figure 2:**
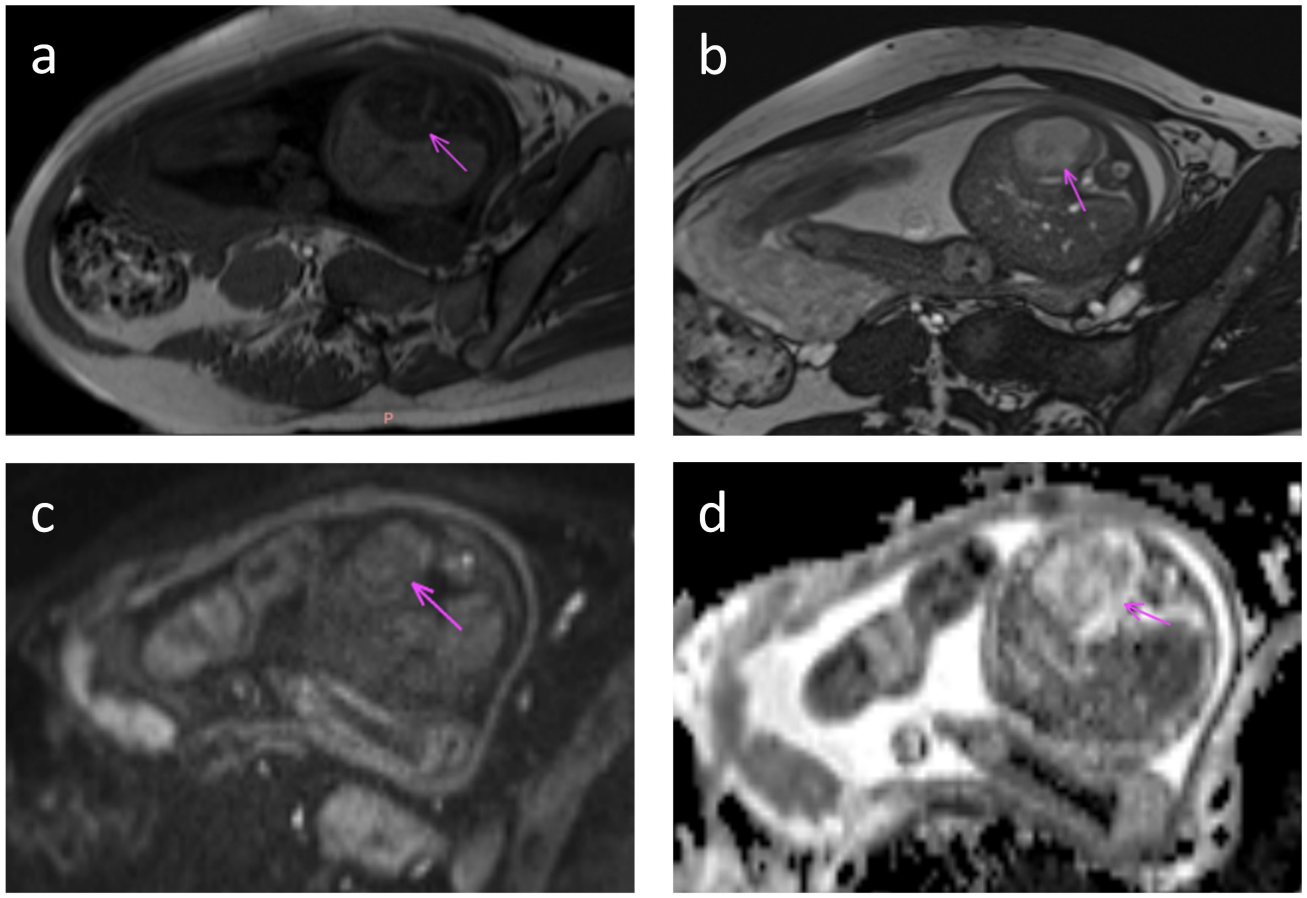
Fetal MRI at 36 weeks gestation. Axial MRI images through the fetal abdomen show a tumor in the left lobe of the liver (arrow). **(a)** T1-weighted image shows low T1 signal in the tumor. **(b)** T2-weighted image shows high T2 signal. **(c)** Diffusion weighted image with b value = 800 shows intermediate signal and **(d)** ADC map shows typical heterogeneous signal with areas of facilitated and restricted diffusion.

Four patients (No.s 4, 5, 7, and 8) underwent prenatal MRI, which revealed solitary well-circumscribed round/ovoid lesions with distinct margins. Compared with normal hepatic parenchyma, all masses demonstrated hypointense signals on T1WI and hyperintense or predominantly hyperintense heterogeneous signals on T2WI. Two cases (No.s 4 and 8) exhibited relatively homogeneous intralesional signals, whereas the remaining two cases (No.s 5 and 7) displayed a heterogeneous internal architecture with dilated intrahepatic vascular channels. Notably, serpiginous flow voids were visualized on T2WI sequences in the latter case, although no significant diffusion restriction was observed on diffusion-weighted imaging (DWI) (**Figure 2**). All four prenatal MRI scans confirmed the absence of intralesional calcifications.

### 3.3 Postnatal follow-up results

**Figure 3:**
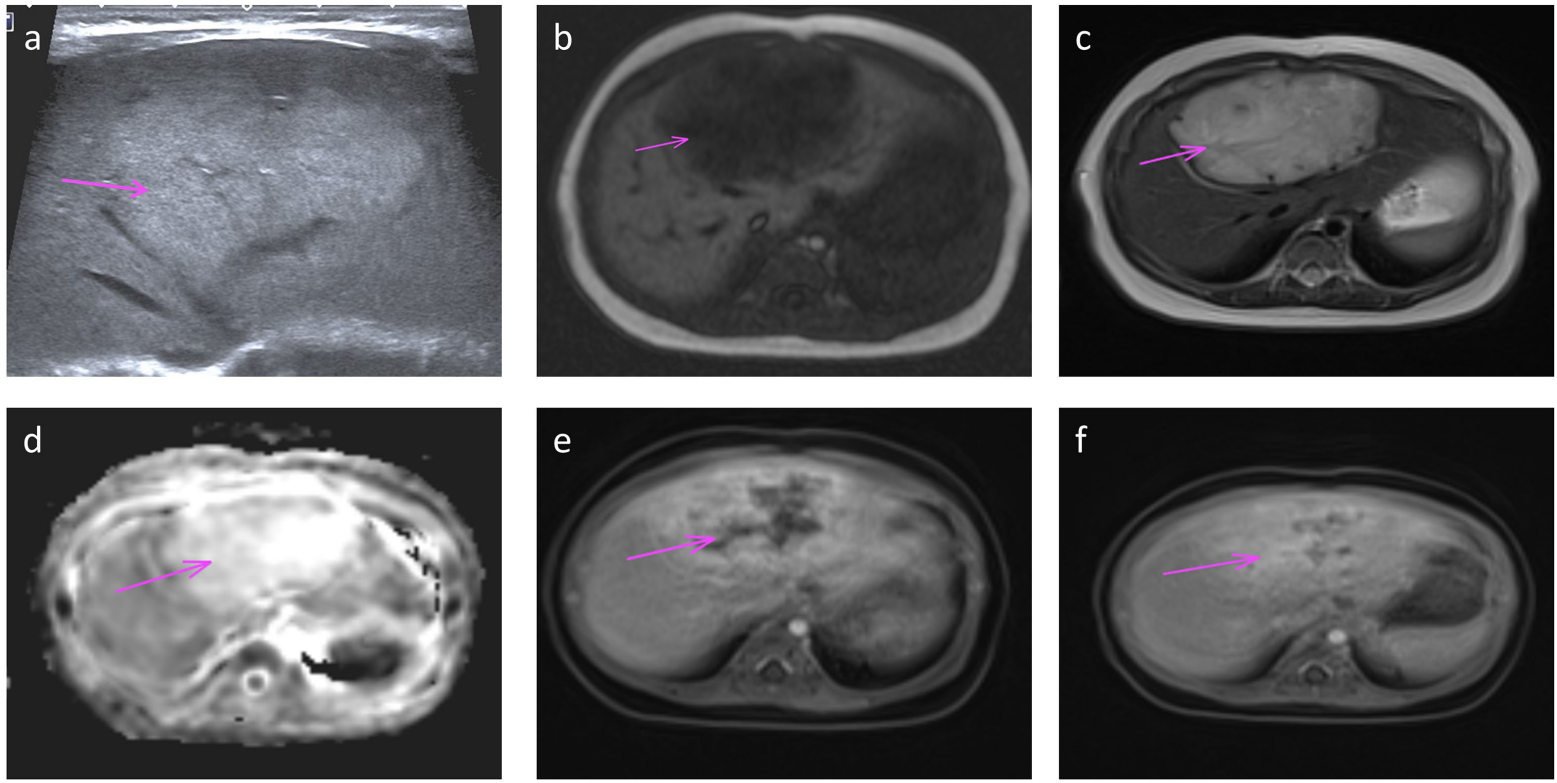
Ultrasound **(a)** and MRI **(b-f)** performed at one month of age shows a large ovoid mass in the left lobe (arrow). Transverse ultrasound image **(a)** of the left lobe shows homogeneous increased echotexture. MR axial T1 and T2 weighted images **(b, c)** show low T1 and high T2 signal with vascular flow voids on T2; high signal on diffusion ADC map **(d)**; and centripetal filling on dynamic-enhanced images with peripheral enhancement in the arterial phase **(e)** and progressive filling in the delayed phase **(f)**.

**Figure 4:**
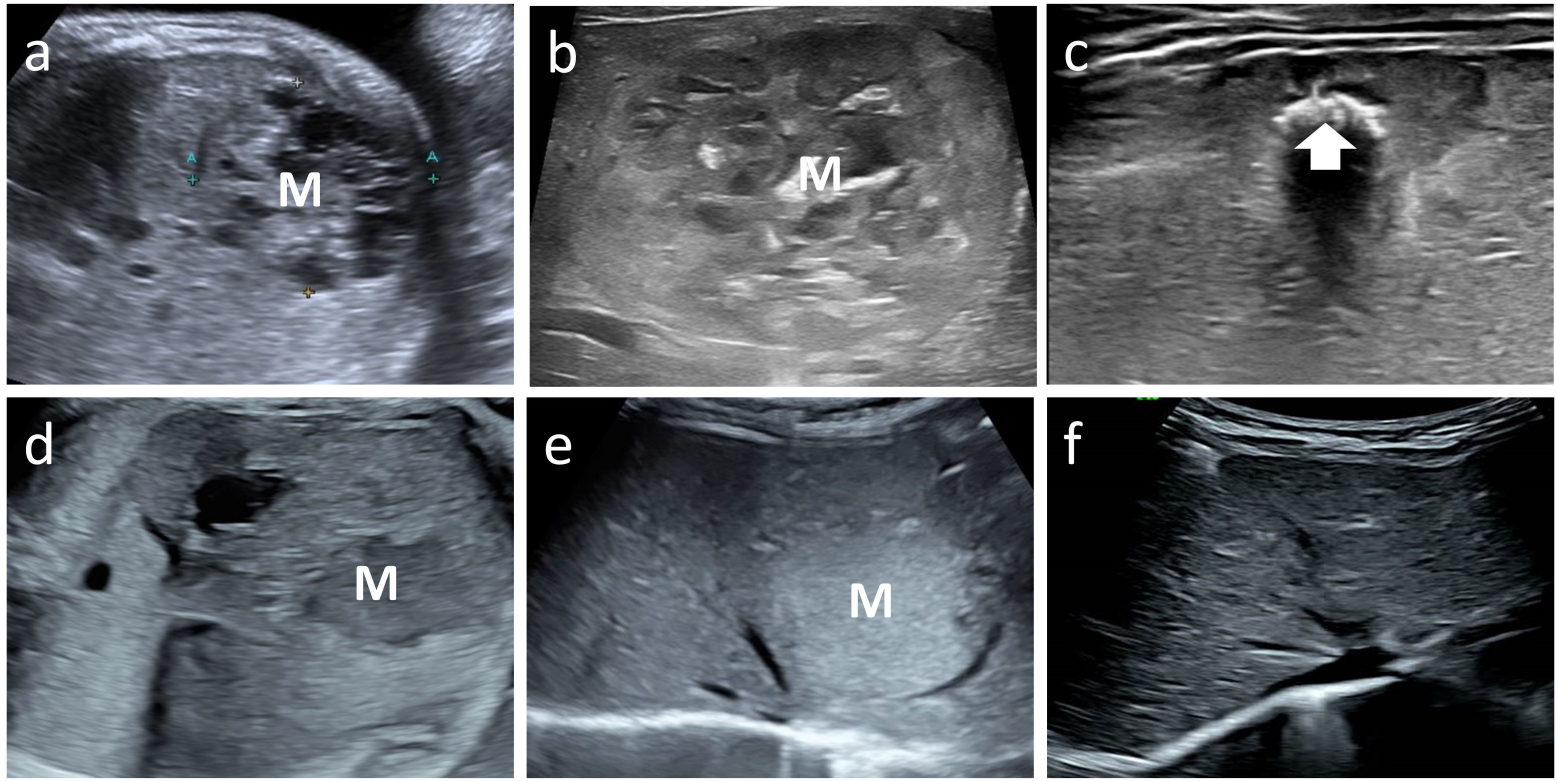
Case 6 and 8 with mass (M) on fetal ultrasound, postnatal ultrasound with maximum volume and follow up image showing regression. Case 6 fetal and postnatal images **(a, b)** show internal heterogeneous, honeycomb hypoechogenicity. The tumor volume peaked at 112 ml. The final regression image **(c)** shows patches of echogenic calcification (arrow). Case 8 fetal and postnatal images **(d, e)** show a homogeneous mass, hypoechoic on prenatal ultrasound and hyperechoic on postnatal ultrasound. The tumor volume peaked at 13 ml. The tumor eventually regressed completely **(f)**.

**Figure 5:**
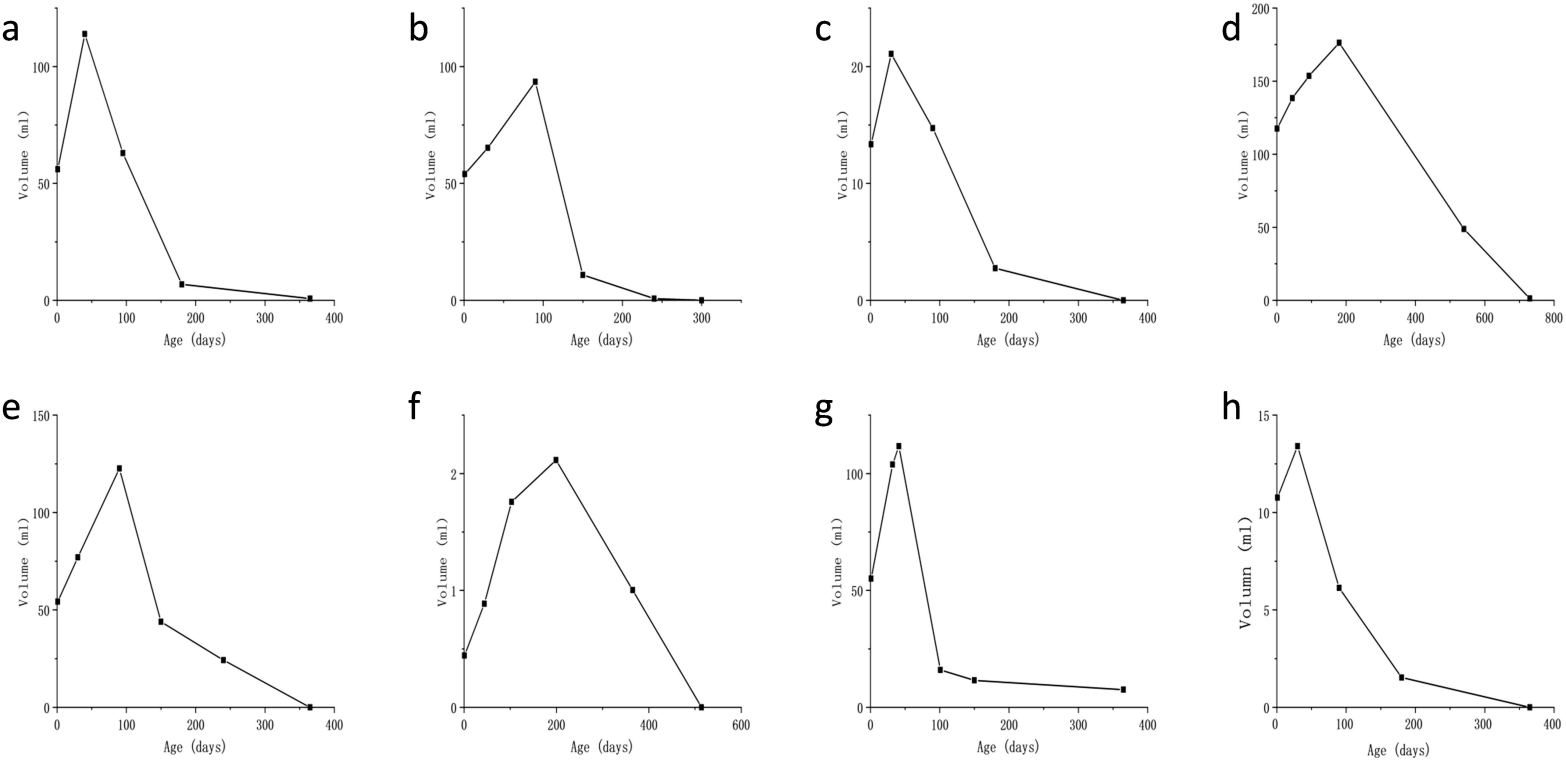
Plot of lesion volume versus time since birth for the eight patients **(a–h)**. The y-axis represents volume (in ml), and the x-axis is time since birth (in days). Graphs **(a–c, e, g, h)** show a sharp increase to a peak around 100 days, then a decline. Graph **(d)** peaks around 200 days, and graph **(f)** peaks later, around 200 days, with lower values.

All patients delivered vaginally at term, with four neonates (50%) being male. At the time of follow-up, none of the patients exhibited clinical symptoms or complications, such as prenatal tumor rupture and bleeding, fetal heart enlargement, atrioventricular regurgitation, fetal anemia, fetal distress *in utero*, high-output heart failure and thrombocytopenia after birth, tumor rupture and bleeding, etc. And their AFP levels, thyroid function tests (TSH and free T4), and fibrinogen levels were within the normal ranges. MR findings in neonates did not significantly differ from those obtained prenatally. Enhancement scans revealed contrast enhancement in the arterial phase, typically demonstrating peripheral nodular distribution, whereas the delayed phase showed a centripetal filling pattern (**Figure 3**). Furthermore, all patients presented with tumors of smaller volume than their initial size, with complete regression observed in six cases and presence of strong echocalcification clusters in two cases (**Figure 4**). The median time to peak tumor size was 66 days (range 30–199 days), with 75% (6/8) of the peak tumor volumes occurring within 100 days. The average maximum tumor volume was 2.14 times the initial volume at birth (range 1.24-4.78 times). Within one year, 87.5% (7/8) of the tumors regressed to 80% of their initial size, with a median time to complete regression of 365 days (range 300–730 days) (**Figure 5**).

## 4 Discussion

We identified a rare type of hepatic hemangioma and summarized its prenatal imaging features and postnatal growth processes. Similar to CHH, they are detected prenatally; however, they undergo rapid proliferation after birth. In this series, the initial volume exhibited wide variation (0.3-46.5 ml), yet demonstrated rapid growth in all cases, with peak volume exceeding 1.5 times the initial volume within the first six months postpartum. Subsequently, the tumors regressed within a few months without any adverse symptoms or complications in any of the patients. Furthermore, the vast majority of tumors showed complete regression within one year of birth. No proliferation after birth has always been regarded as a characteristic of CHH (6). In recent years, some experts have reported CHH that continues to proliferate after birth (15), and our research has further confirmed the existence of the fourth subtype.

Prenatal US is the primary imaging modality used to detect fetal structural anomalies. In systematic prenatal examinations, fetal hepatic masses demonstrate a relatively high detection rate, enabling a comprehensive evaluation of lesion characteristics, including location, morphology, dimensions, vascularity, echogenicity, and spatial relationships with adjacent tissues. Furthermore, US effectively identifies associated complications such as fetal hydrops, cardiac dysfunction, and amniotic fluid volume abnormalities. Its excellent reproducibility makes it suitable for the longitudinal monitoring of hepatic hemangiomas throughout gestation (4). However, this technique has inherent limitations, including suboptimal soft tissue contrast resolution and a restricted field of view, with operator-dependent diagnostic accuracy influenced by maternal adiposity, fetal positioning, and amniotic fluid volume. Dong et al. (3) suggested that complementary MRI evaluations during pregnancy enhanced the diagnostic assessment of hepatic neoplasms. MRI offers superior soft tissue contrast resolution, facilitating detailed analysis of intralesional signal characteristics and vascular infiltration patterns. Postnatally, contrast-enhanced MRI provides additional diagnostic value by demonstrating temporal variations in vascular perfusion, thereby enabling the differentiation between CHH and hepatoblastoma, a distinction that is often challenging with US alone (16).

The prenatal imaging characteristics of this type of hepatic hemangioma closely resemble those previously described in CHH. The ultrasonographic features include a predilection for late pregnancy, focal lesions with well-defined margins, a predominantly hypoechoic interior, and tortuous vascular patterns. The tumor exhibited relatively abundant blood flow signals, with visible portal vein supply, hepatic venous drainage, and occasional co-supply by the hepatic artery. The peripheral blood flow manifests as a circular or semicircular pattern (2, 4, 14). The MRI-visible lesion demonstrated a classically round and well-defined hyperintense signal on T1WI and T2WI, with no apparent diffusion restriction on DWI. Vascular voids were occasionally observed within the lesions. Postnatal MR enhancement scans typically revealed an early peripheral enhancement pattern characterized by a nodular distribution of contrast enhancement, followed by a centripetal filling pattern in the delayed phase (2, 17, 18). Additionally, our findings indicate that larger tumors often display heterogeneous honeycomb-like echoes within their interior and exhibit higher vascular density. This observation aligns with a previous study on CHH, which noted the uniformity of smaller vascular tumors and an increased likelihood of heterogeneity in larger tumors (6).

The presence of calcification in a prenatal tumor is often considered a specific sign of CHH and can be used to distinguish it from other prenatal lesions. However, this type of tumor does not exhibit calcification during prenatal and postnatal growth but becomes apparent as the tumor begins to shrink and increases in size with the degree of degeneration. The presence of calcification in the tumor during the pathological process is considered a characteristic of the remission period in CHH (2, 19). This also indicates an active proliferation stage for this type of HH before birth. Additionally, there were no venous lakes, intratumor hemorrhages, or abnormal vascular shunts within the tumors of these patients. Based on previous research (2, 8), the presence of venous lakes or arteriovenous shunts within the tumor is considered a negative prognostic factor, potentially leading to adverse postnatal outcomes, such as congestive heart failure in infants. Therefore, the imaging findings suggest that this hepatic hemangioma developed incompletely *in-utero* and is expected to continue proliferating after birth, with a favorable prognosis.

Prior to this, in previous literature (4, 20), CHHs were predominantly located in the right lobe of the liver. However, our study revealed a significant predilection for the left lobe in this series of hepatic hemangiomas, with only one instance found in the right lobe. Nevertheless, owing to the limited sample size, it is challenging to ascertain whether genuine left-lobe predominance exists.

Following postnatal imaging, the tumor exhibited unrestricted diffusion on DWI and demonstrated gradual nodular enhancement in the peripheral area during the arterial phase, followed by centripetal filling in the delayed phase. Additionally, all AFP test results remained within the normal range throughout the follow-up period. These findings excluded the possibility of congenital hepatoblastoma (16).

According to the Liver Hemangioma Registry classification, liver hemangiomas are categorized as focal, multicentric, and diffuse. Focal hemangiomas are hepatic manifestations of congenital hemangiomas, whereas multicentric and diffuse types are associated with infantile hepatic hemangiomas (IHH) (21). IHH is typically undetectable before birth, manifests within the first two weeks after delivery, and is potentially accompanied by cutaneous IH (22). Throughout the regression process, thrombosis, calcification, or necrosis were absent (23). Furthermore, the cases examined in this study involved localized lesions that presented prenatally without concurrent skin manifestations. In contrast to IHH, which exhibits a higher incidence in females and may be associated with exocrine hypothyroidism in patients (24), these hepatic vascular tumors and CHH share similarities in lacking a sex predilection or ethnic bias, all of which exhibit normal thyroid function. Consequently, it is more appropriate to classify these liver vascular tumors as CHH rather than as IHH.

The overlap in both pathological and genetic features between IHH and CHH, as well as among the three types of CHH (1, 5, 25, 26), poses challenges in predicting growth trends based on imaging findings. The specific classification of these tumors can only be determined through follow-up observations of tumor changes and clinical progression in affected children. It is quite possible that a new type of tumor lies between them, such as the 11 cases of delayed expansion of congenital cutaneous vascular tumors reported by Chen Hua (27). Consequently, we believe that this potential type is more likely to have resulted from random selection. In addition, the report by Gong X et al. (15) mentioned two different outcomes of postnatal proliferation of CHH, one was complete regression after proliferation, and the other was partial regression after proliferation. This may be due to the fact that their study was a multi-center study with a large sample.

This study had two primary limitations. First, there is a lack of pathological examination results, which reflects the clinical reality that imaging results are usually sufficient for diagnosis and follow-up and that most lesions do not require biopsy. Second, although the number of cases was limited, this retrospective study is the sole report on the prenatal characteristics and postpartum growth patterns of hepatic hemangiomas of this type. We intend to continue collecting such cases for further statistical analyses.

In conclusion, we delineated a distinct subtype of CHH characterized by unique clinical manifestations that set it apart from the previously classified subtypes. This type of CHH does not show calcification in the tumor before birth, and the tumor continues to proliferate after birth; however, with the passage of time, the tumor spontaneously regresses without complications. Most of the patients experienced rapid regression (within 18 months). Therefore, to monitor the postnatal growth of CHH, we recommend regular imaging observations that do not interfere with the normal growth of children. This approach aims to prevent misdiagnosis as malignant tumors and avoid unnecessary drug treatments or surgical interventions.

## Data Availability

The raw data supporting the conclusions of this article will be made available by the authors, without undue reservation.
